# Age-Related Macular or Retinal Degeneration?

**DOI:** 10.3390/medicina59050920

**Published:** 2023-05-11

**Authors:** Michalina Gałuszka, Dorota Pojda-Wilczek, Izabella Karska-Basta

**Affiliations:** 1 Department of Ophthalmology, Faculty of Medical Sciences in Katowice, Medical University of Silesia, 40-514 Katowice, Poland; 2 Kornel Gibiński University Clinical Centre, 40-514 Katowice, Poland; 3 Jagiellonian University Medical College, Faculty of Medicine, Department of Ophthalmology, Division of Ophthalmology and Ocular Oncology, 31-501 Kraków, Poland

**Keywords:** age-related macular degeneration, maculopathy, choroidal neovascularization, electrophysiology, electroretinography, full-field electroretinography

## Abstract

Age-related macular degeneration (AMD) is an eye disease that leads to progressive vision loss. Its prevalence has been increasing due to population aging. Previously, it was commonly believed that the disease affects the central retina, that is, the macula. However, recent studies have shown that it also involves the peripheral retina. Novel imaging techniques revealed various degenerative lesions that extend beyond the central macula. While their prevalence remains unknown, they seem to be more frequent in patients with late AMD. These findings suggest that the term “age-related retinal dysfunction” might be more adequate to describe some cases of AMD. They also raise the question about the role of electroretinography (ERG) as an objective measure of retinal function. The most common types of ERG tests used in AMD are multifocal (mfERG) and full-field ERG (ffERG). mfERG is more sensitive to macular changes, but the test is difficult to perform when fixation is unstable. On the other hand, ffERG reflects the function of the entire retina, not only the macular area. It helps assess the impact of peripheral retinal lesions and overall retinal function in patients with AMD. As ffERG results are normal in early-stage AMD, any abnormalities indicate that the disease is more severe and affects the entire retina. Anti-vascular endothelial growth factor injections improve retinal function in patients with neovascular AMD, as demonstrated by an increase in their ERG responses. More research is needed to assess the association between local and general retinal dysfunction. In this review, ffERG findings in patients with AMD are described and the usefulness of ffERG is discussed based on previous studies and cases from our own clinical practice.

## 1. Introduction

Age-related macular degeneration (AMD) is the most common cause of progressive vision loss in developed countries. Moreover, its prevalence is likely to increase due to population aging [1]. AMD was described as an exaggerated form of retinal aging, compared to a physiological decline in retinal function observed in elderly people [2]. Early dry AMD is characterized by the presence of soft drusen and changes in the Bruch’s membrane, choriocapillaris, retinal pigment epithelium (RPE), and photoreceptors. On the other hand, the characteristic features of late dry AMD are geographic atrophy, epithelial pigment detachment, subretinal neovascularization, hemorrhage, and fibrous scar. The prevalence of early AMD was reported to be 8% among individuals aged 45 years or older, compared with 0.4% for late AMD [1]. Neovascular (wet) AMD is used to describe macular degeneration with choroidal neovascularization (CNV). This type affects about 10% of AMD patients but accounts for about 90% of cases of severe vision loss associated with the disease [3].

## 2. Retinal Function Assessment

Electroretinography (ERG) is an objective measure of retinal function, in which changes in electrical polarization are graded depending on the duration and intensity of the light stimulus [2]. Rod-driven and cone-driven retinal responses can be differentiated based on specific light and dark adaptation levels [2,3]. Full-field ERG (ffERG) is a type of ERG that facilitates diagnosis and prognosis assessment in retinal disease. It measures an electrical response from the entire retina, and not just the macular area. Moreover, in patients with localized retinal disease, ffERG results are normal, as reported for various macular diseases [4]. Therefore, the diagnostic test of choice in patients with macular disease is multifocal ERG (mfERG). Numerous studies have confirmed that mfERG is a useful tool for monitoring the disease and treatment outcomes [5].

**Figure 1:**
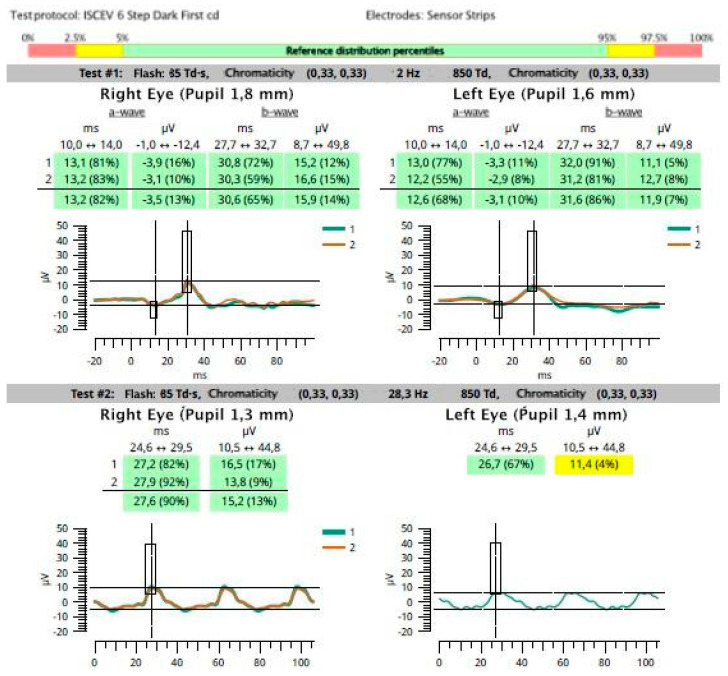
A 70-year-old man with late atrophic age-related macular degeneration of the right eye (RE) and anisometropic amblyopia of the left eye (LE). Best corrected visual acuity (BCVA; Snellen chart): RE = 0.05 (correction +4.0 spherical diopters), LE = 0.2 (correction +3.25 spherical diopters and +3.0 cylindrical diopters in the 90-degree axis). Photopic ffERG (RETeval, LKC Technologies, Gaithersburg, MD, USA, sensor strip electrodes) in both eyes within normal limits. In this case, retinal degeneration is limited to the macular region.

An example of normal photopic ffERG response in a patient with late wet AMD in the right eye, and low visual acuity in the fellow eye caused by anisometropia (anisometropic amblyopia), is presented in Figure 1.

## 3. Peripheral Retina in Multimodal Imaging

**Figure 2:**
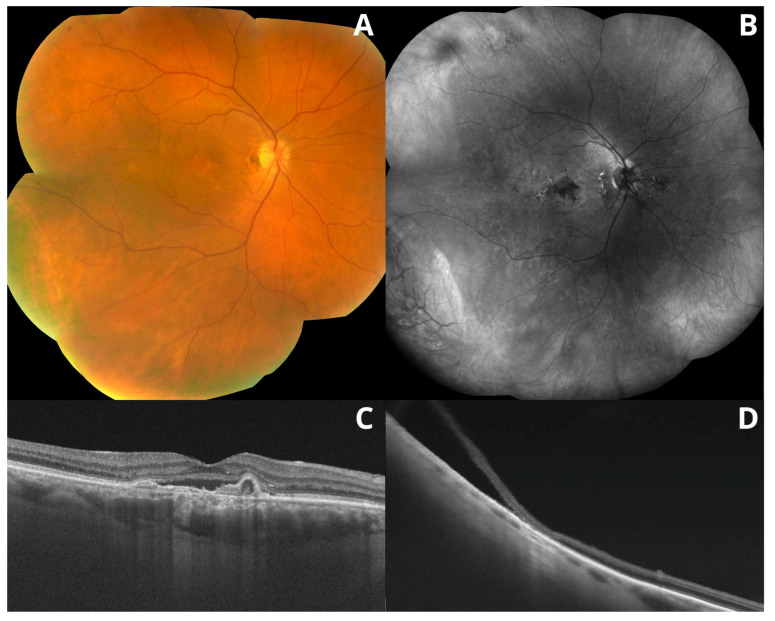
Multimodal imaging of the retina. Color fundus image (**A**) and fundus autofluorescence (**B**) (FAF; Heidelberg Spectralis HRA, Heidelberg, Germany). FAF revealed atrophic RPE lesions, which are not visible in a color fundus image. Optical coherence tomography (Topcon DRI OCT Triton Imagenet, Tokyo, Japan) revealed peripheral degenerations and retinoschisis (**D**) and age-related macular degeneration (**C**).

**Figure 3:**
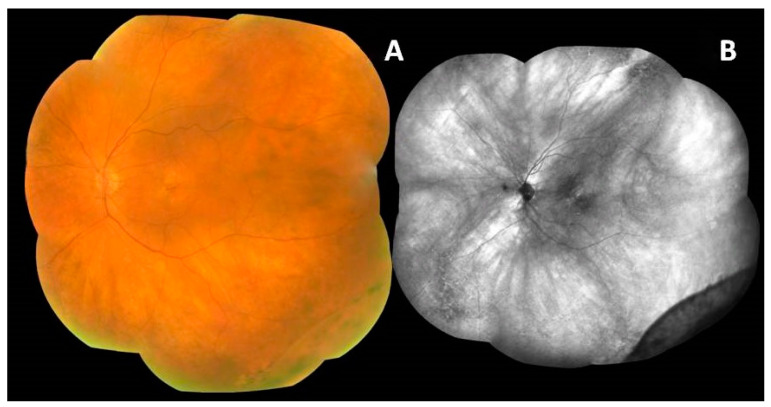
Age-related macular degeneration and peripheral retinoschisis. Fundus autofluorescence (**B**) highlights atrophic lesions and the border of peripheral retinoschisis seen in a color image (**A**).

**Figure 4:**
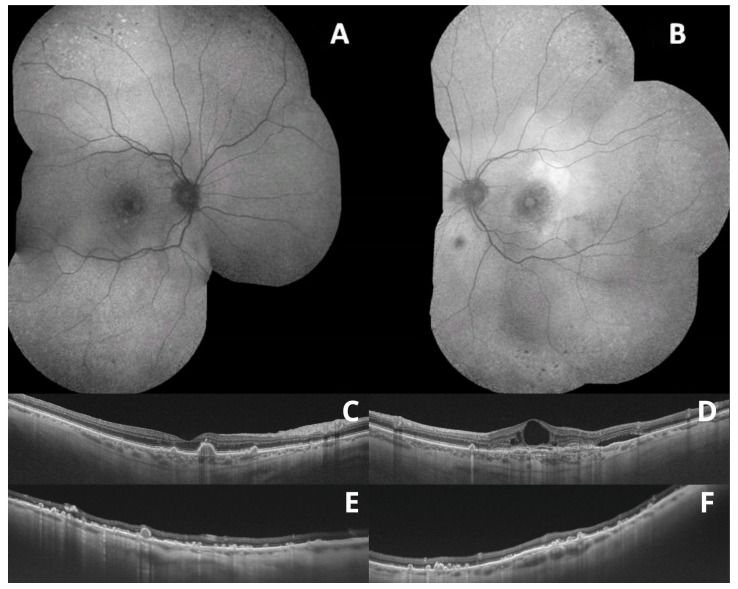
Wide-field fundus autofluorescence showing peripheral degenerations (**A**,**B**); optical coherence tomography scans showing macular drusen (**C**,**D**); and peripheral drusen (**E**,**F**).

So far, AMD has been generally considered a disease of the central retina, that is, the macula. However, recent studies have shown that, apart from the macular area, AMD may also affect the peripheral retina. Several studies have confirmed that peripheral retinal lesions are more common in patients with AMD than in healthy individuals, as assessed by ultrawide-field imaging and optical coherence tomography [1,4,6]. Moreover, these changes seem to be more frequent in eyes with late AMD (neovascular AMD and/or geographic atrophy). However, the relevance of these peripheral lesions has not been elucidated [2,7]. Example images of peripheral retinal degeneration and retinoschisis in patients with AMD are shown in Figure 2 and Figure 3. Peripheral drusen and perivascular pigmentation are depicted in Figure 4.

Novel imaging techniques reveal that peripheral retinal lesions can co-occur with macular disease. The most useful tools are wide-field scanning laser ophthalmoscopy, digital wide-field angiography, and wide-field fundus autofluorescence [8].

Borooah et al. [9] used multimodal imaging to evaluate the natural history of reticular pseudodrusen (RPD) in patients with late-onset retinal degeneration. Some clinical features of this rare inherited disease mimic those seen in AMD, and RPD are considered to be a risk factor for AMD progression to geographic atrophy. Reticular pseudodrusen occur between the neural retina and the RPE, and can be found from the perifovea to the retinal periphery. Based on ffERG findings, this study confirmed rod dysfunction associated with RPD. In patients with AMD, RPD may cause abnormal night adaptation. The investigators concluded that patients with AMD should be followed-up with to assess the progression of RPD [9].

Corbelli et al. [10] used multimodal imaging to assess drusen in patients with and without AMD (mean age, 77 years). They reported AMD in about 54% of eyes with peripheral drusen. However, no association between the clinical features of AMD and peripheral drusen was found. According to the authors, peripheral drusen was a distinct entity rather than the peripheral manifestation of AMD [10].

Sabeti et al. [11] reported a positive correlation between retinal sensitivity and retinal thickness outside of the macula. They concluded that outer macular thickness and retinal sensitivity, together with visual acuity, may have a better prognostic value in patients with AMD than visual acuity alone.

## 4. Function of the Peripheral Retina in AMD

Some investigators reported that ffERG is useful for assessing the impact of peripheral retinal lesions and helps us to understand overall retinal function among patients with AMD. Forshaw et al. [2] performed ffERG in patients with AMD using a standard protocol as per the International Society for Clinical Electrophysiology of Vision (ISCEV) guidelines. They found no significant differences in dark-adapted ffERG between patients with early AMD and individuals without vitreoretinal disease (controls). The light-adapted 3.0 a-wave and b-wave implicit times were similarly prolonged in patients with early AMD and the healthy controls. Moreover, there were no significant differences in the 30-Hz flicker peak time between patients with early AMD and the controls. A linear regression analysis revealed that functional changes in early AMD reflect the normal aging of the retina. On the other hand, patients with late AMD had a prolonged light-adapted 3.0 a-wave implicit time and 30-Hz flicker peak time on ERG, as compared with the controls. This indicates damage of the cone system beyond the macula in late AMD. Prolonged implicit times on ERG are a well-known indicator of progressive and more generalized disease. Moreover, rod-driven b-wave implicit times are longer in patients with geographic atrophy, as compared with those in other AMD populations [2].

Jackson et al. [12] investigated the impact of aging and AMD on the activation of phototransduction in rod photoreceptors by measuring the a-wave of ffERG in 39 older adults with early AMD and 41 individuals with normal retinal health who served as controls. For comparison purposes, 27 young adults were also enrolled. Additionally, rod-mediated ERG parameters were compared between phakic and pseudophakic eyes. Dark-adapted responses did not differ significantly between phakic and pseudophakic groups. The investigators concluded that the prolonged rod a-wave implicit time in phakic eyes may be due to lens aging and their increased density and light absorption, rather than due to retinal aging. Thus, the utility of ffERG in the early diagnosis of AMD is limited, and the lens status should be considered [12].

While the cones are localized mostly in the macula, the rods are common across the whole retina [3]. There are nearly 100 million rod photoreceptors and only 4.5 to 6.0 million cones in the retina. Ninety percent of the rods are localized in the peripheral retina, outside the macula [13]. The retinal periphery is used for night vision, movement detection, and locomotion and postural stability; therefore, its function is relevant to the vision-related quality of life of elderly people. This is especially important for patients with central vision loss, such as those with AMD, because they depend on their peripheral retinal function for everyday activities [14].

Owsley et al. [15] reported that prolonged rod-mediated dark adaptation corresponding to the early damage of rod photoreceptors is a functional biomarker of early AMD.

Ronan et al. [16] studied senile panretinal cone dysfunction in patients with any type of AMD versus age-matched controls. The investigators found that light-adapted b-wave amplitudes were significantly lower in patients with AMD. Photopic a-wave implicit time differed significantly between groups with geographic atrophy or drusen and geographic atrophy with CNV. The results suggest that CNV causes not only local, but also more advanced abnormalities in the entire retina [16]. Walter et al. [17] reported that both light-adapted a-wave and b-wave amplitudes were significantly reduced in patients with AMD compared with healthy, age-matched controls. Moreover, light-adapted a-wave implicit times were significantly prolonged in AMD patients, while b-wave implicit times were comparable with those in the controls. Dark-adapted a-wave and b-wave amplitudes were significantly reduced, and implicit times were significantly prolonged, in patients with AMD versus the controls [17].

Dimopoulos et al. [3] used ffERG to examine patients with wet AMD in one eye and dry AMD in the fellow eye. They reported abnormalities in both eyes, including prolonged implicit times of dark-adapted a-wave and b-wave. No differences in implicit times in light-adapted response, or in amplitude in dark-adapted and light-adapted responses, were found. It is possible that the implicit time prolongation might predict the onset of CNV in the fellow eye [3]. CNV should be regarded as an advanced stage of retinal degeneration.

**Figure 5:**
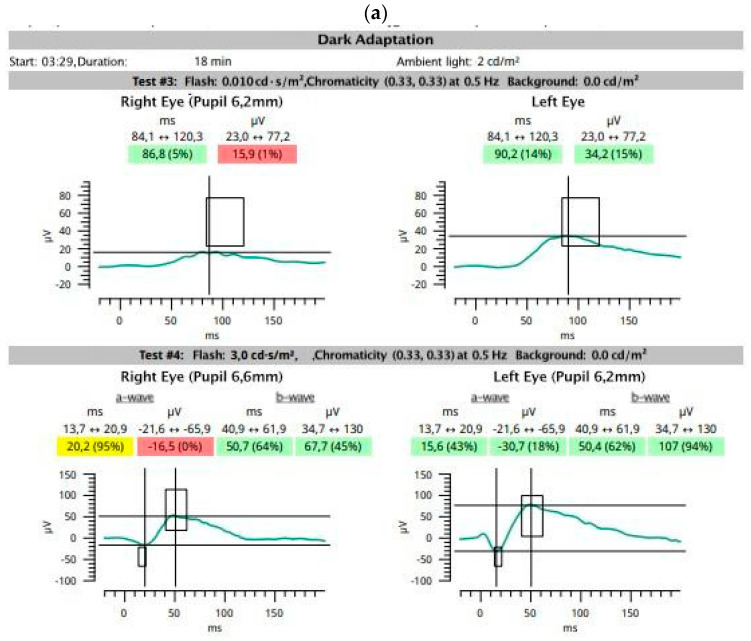

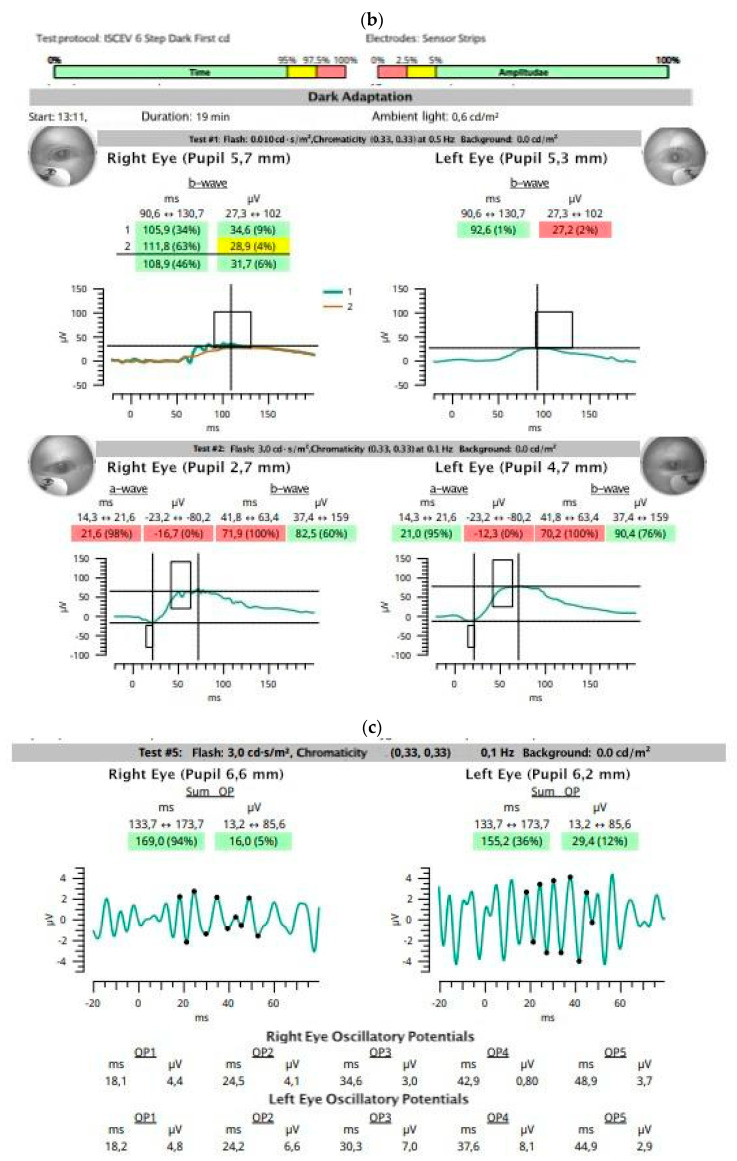

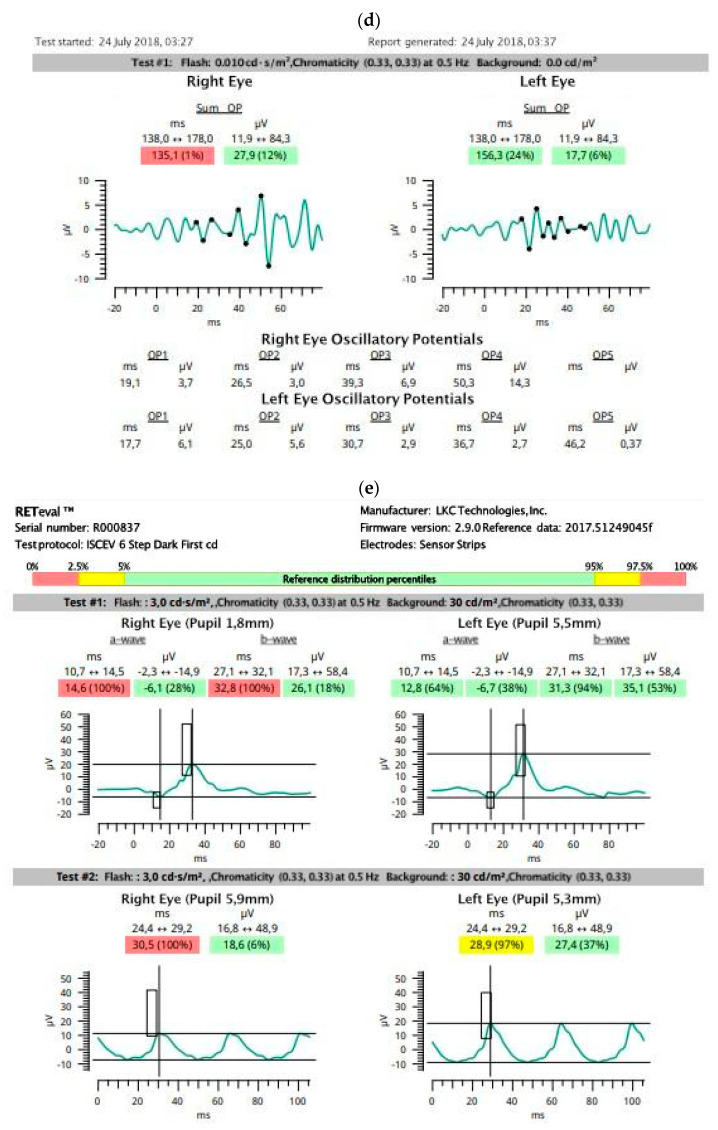

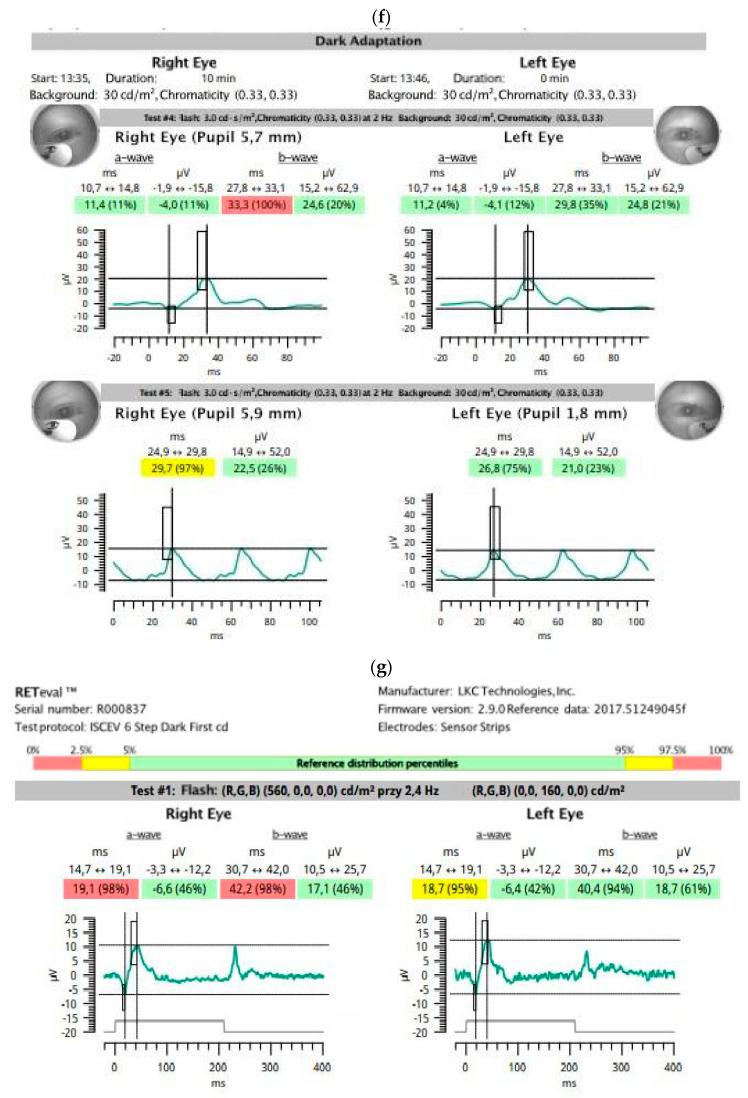

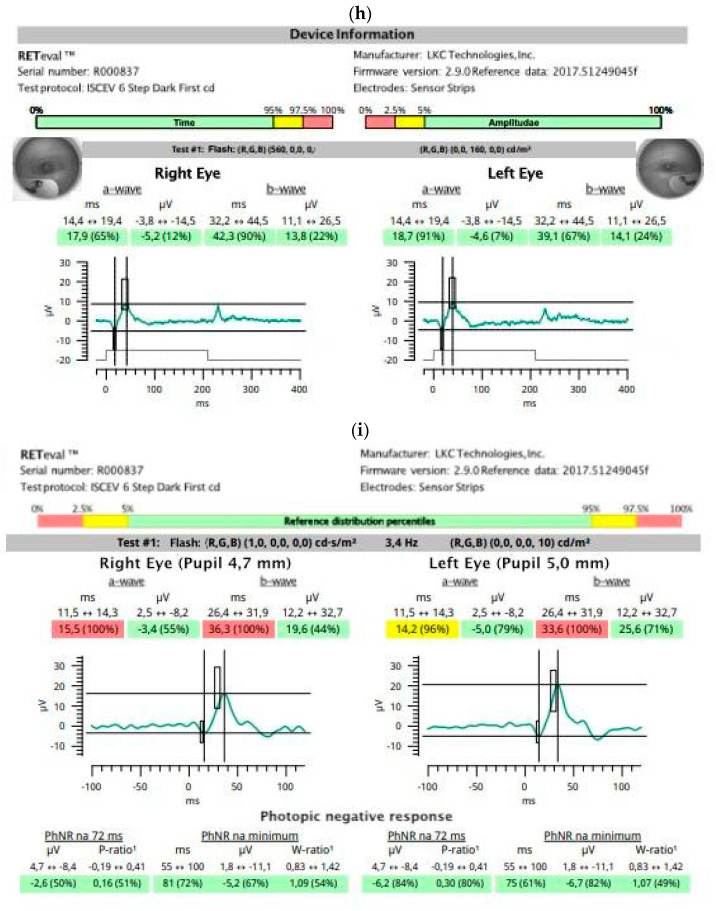

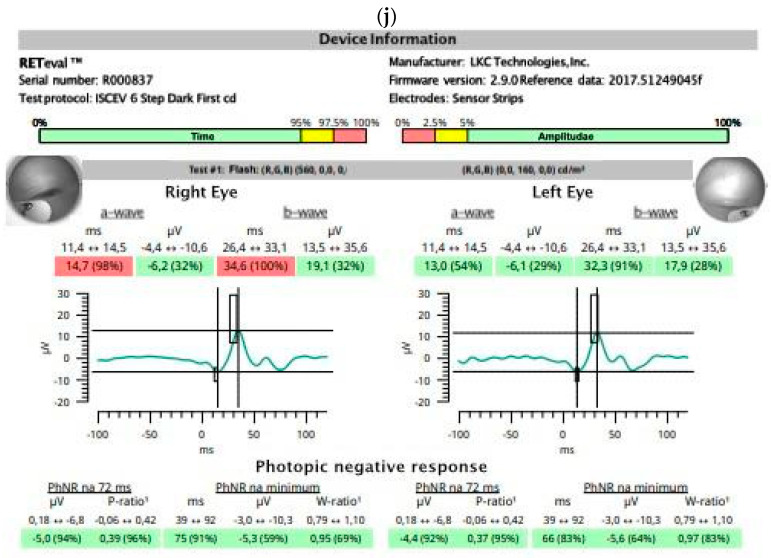
A 74-year-old woman with neovascular age-related macular degeneration (AMD) of the right eye (RE) and dry AMD with numerous drusen in the macula of the left eye (LE). Previously, macular degenerations were similar in both eyes. Best corrected visual acuity (BCVA): RE = 0.1 and LE = 1.0. ffERG (RETeval, LKC Technologies, Gaithersburg, MD, USA, sensor strip electrodes) before and after 4 anti-vascular endothelial growth factor injections into the RE. Follow-up at 8 months. BCVA after treatment: RE = 0.125 and LE = 1.0. Scotopic ffERG was abnormal in the RE before treatment (**a**) with little improvement after treatment (**b**). The amplitudes of oscillatory potentials were low in both eyes before and after treatment (**c**,**d**). On photopic ffERG, markedly prolonged a-wave and b-wave implicit times were noted in the RE; a-wave and b-wave amplitudes were low in the RE before treatment (**e**) and were low in both eyes at follow-up (**f**). After treatment, the amplitudes became higher in the treated eye and lower in the fellow eye. The function of ON-center and OFF-center bipolar cells was similar in both eyes before treatment (**g**) and did not change after treatment (**h**). The photopic negative response revealed normal retinal ganglion cell function in both eyes before (**i**) and after (**j**) treatment. ffERG findings were abnormal in both eyes, but a significantly prolonged implicit time of a-wave and b-wave was noted in the RE only.

Example ffERG images of photopic retinal function in both eyes are shown in Figure 5. In the eye with CNV, implicit time was significantly prolonged. The most important data presented in Figure 5 were additionally compared in Table 1. Follow-up examination revealed some improvement in retinal function in the treated eye and deterioration in the fellow eye.

Forshaw et al. [4] conducted a systematic review and meta-analysis of studies on ffERG in patients with AMD and healthy controls. They reported reduced a-wave and b-wave amplitudes in dark-adapted and light-adapted responses irrespective of the type of AMD. In light-adapted responses, a delay of a-wave and b-wave implicit times was noted. Significant abnormalities in retinal function were found in late, but not in early, AMD. In patients with early AMD, retinal function was comparable to that of the healthy controls [4].

Mackay et al. [18] compared the results of various ERG types (pattern, photopic full-field, multifocal) between eyes with neovascular AMD and fellow eyes. The amplitudes of all analyzed parameters were lower in eyes with AMD, while only slight differences were found in implicit time.

In their study, Skaat et al. [19] reported a significant improvement in ffERG a-wave amplitude responses in patients with neovascular AMD under both scotopic (representing rod function) and photopic (representing cone function) conditions, even after a single intravitreal injection of anti-vascular endothelial growth factor (anti-VEGF). They linked this to improved photoreceptor function in these patients. By inhibiting the activity of the vascular endothelial growth factor in the retina and the choroid, anti-VEGF therapy may reduce vessel transmission and support reabsorption, thereby further improving photoreceptor function. In addition, anti-VEGF agents may enhance photoreceptor degradation. All of these effects contribute to an improvement in a-wave response on ERG after intravitreal anti-VEGF injection.

Nishimura et al. [20] performed photopic ffERG and mfERG in patients with AMD before and after intravitreal anti-VEGF injections. They did not report significant differences in ffERG results after therapy, but there were improvements in central vision and the anatomical structure of the macula. On the other hand, mfERG results significantly improved. These findings indicate that anti-VEGF injections did not have any adverse effects on any of the retinal layers, including the cone photoreceptors, cone bipolar cells, amacrine cells, and retinal ganglion cells in the cone-mediated pathway.

Currently, a protocol for ERG developed by the ISCEV makes it possible to compare different results from around the world [21]. The most recent clinical reports add to the current knowledge of impaired overall retinal response in patients with AMD. So far, no standard ERG protocol has been developed specifically for this patient population. While mfERG is sensitive to macular changes, it is more difficult to perform in patients with low visual acuity because it requires stable fixation. As for ffERG, it may show no abnormalities in the early stage of the disease, but any abnormal findings indicate that the disease is more advanced and affects the entire retina [22].

The most recent studies on oxidative stress and mitochondrial damage in the RPE may help elucidate the pathogenesis of AMD and change the diagnostic and therapeutic approach to the disease [23]. The impaired function of the RPE leads to abnormalities in photoreceptors. Photoreceptor dysfunction or loss causes secondary alterations to the remaining retinal cells [24]. This mechanism may be responsible for progressive retinal degeneration in rod and cone dystrophies. The dystrophy usually starts in a certain group of cells. With time, it becomes more widespread, ultimately leading to blindness in most patients.

In summary, the majority of patients with AMD present with morphological changes that are centrally located and do not involve the peripheral retina. However, in some cases, the changes may extend beyond the central macula. These findings suggest that the term “age-related retinal dysfunction” might be more precise to describe the disease entity. Further studies are needed to assess the relationship between local and general retinal dysfunction and treatment outcomes in patients with neovascular AMD. In our opinion, ERG, and especially ffERG, should be performed before and after anti-VEGF injections in order to determine retinal response and assess retinal function during anti-VEGF therapy for AMD. ERG should be performed on both eyes at the same time and under the same conditions, allowing the untreated fellow eye to be used as a control. With such an approach, even small changes might possibly be revealed. Moreover, it might be useful in identifying any potential prognostic factors for patients undergoing intravitreal anti-VEGF therapy.

## 5. Conclusions

Evidence of retinal function impairment on ffERG indicates that AMD is not only a macular disease but also affects the peripheral retina, especially in later stages of the disease. Studies on larger populations and with a longer follow-up are needed to assess ffERG data in combination with multimodal wide-field imaging of the retina. The use of extended ISCEV protocols and other functional tests may allow for the further assessment of retinal dysfunction in eyes with AMD.

## Figures and Tables

**Table 1:** Summary of the most important numeric data presented in Figure 1 and Figure 5: electroretinography results before and after treatment of the right eye with age-related macular degeneration.

	Step	DA 0.01		DA 3.0				DA 3 OPs		LA 3				LA 30 Hz		PhNR	
	Wave	b	b	a	a	b	b	OP	OP	a	a	b	b	b	b	Minimum	
	Eye	T, ms	A, µV	T, ms	A, µV	T, ms	A, µV	T, ms	A, µV	T, ms	A, µV	T, ms	A, µV	T, ms	A, µV	T, ms	A, µV
Figure 1	AMD									13.2	−3.5	30.6	15.9	27.6	15.2		
	FE									12.6	−3.1	31.6	11.9	26.7	11.4		
Figure 5a,c,e,i	AMD	86.8	15.9	20.2	−16.5	50.7	67.7	169.0	16.0	14.6	−6.1	32.8	26.1	30.5	18.6	81.0	−5.2
	FE	90.2	34.2	15.6	−30.7	50.4	107	155.2	29.4	12.8	−6.7	31.3	35.1	28.9	27.4	75.0	−6.7
Figure 5b,d,f,j	AMD	108.9	31.7	21.6	16.7	71.9	82.5	135.1	27.9	11.4	−4.0	33.3	24.6	29.7	22.5	75.0	−5.3
	FE	92.6	27.2	21.0	−12.3	70.2	90.4	156.3	17.7	11.2	−4.1	29.8	24.8	26.8	21.0	66.0	−5.6
2.5th percentile		84	23	13	−21	40	34	133	11	10	−2	27	15	24	14	55	−3
97.5th percentile		130	102	21	−80	63	159	178	85	14	−16	33	62	29	52	100	−10

A—amplitude; AMD—eye with age-related macular degeneration; DA—dark adapted; FE—fellow eye; LA—light adapted; OP—oscillatory potential; PhNR—photopic negative response; T—peak time.

## Data Availability

Not applicable.
